# An Ultra-Compact 28 GHz Arc-Shaped Millimeter-Wave Antenna for 5G Application

**DOI:** 10.3390/mi14010005

**Published:** 2022-12-20

**Authors:** Praveen Kumar, Tanweer Ali, Om Prakash Kumar, Shweta Vincent, Pradeep Kumar, Yashwanth Nanjappa, Sameena Pathan

**Affiliations:** 1 Department of Electronics and Communication Engineering, Manipal Institute of Technology, Manipal Academy of Higher Education, Manipal 576104, India; 2 Department of Mechatronics, Manipal Institute of Technology, Manipal Academy of Higher Education, Manipal 576104, India; 3 Discipline of Electrical, Electronic and Computer Engineering, University of KwaZulu-Natal, Durban 4041, South Africa; 4 Department of Information and Communication Technology, Manipal Institute of Technology, Manipal Academy of Higher Education, Manipal 576104, India

**Keywords:** arc-shaped, 5G, millimeter-wave, ultra-compact

## Abstract

The 5th generation (5G) network was planned to provide a fast, stable, and future-proof mobile communication network to existing society. This research presents a highly compact arc shape structure antenna resonating at 28 GHz for prospective millimeter-wave purposes in the 5G frequency spectrum. The circular monopole antenna is designed with a radius of 1.3 mm. An elliptical slot on the radiating plane aids in achieving an enhanced bandwidth resonating at the frequency of 28 GHz. Including an elliptical slot creates new resonance and helps improve the bandwidth. The antenna has an ultra-compact dimension of 5 × 3 × 1.6 mm^3^, which corresponds to an electrical length of 0.46λ × 0.28λ × 0.14λ, where λ is free space wavelength at the resonant frequency. The projected antenna has an impedance bandwidth of 15.73 % (25.83–30.24 GHz). The antenna has a good radiation efficiency of 89%, and the average gain is almost 4 dB over the entire impedance bandwidth. The simulated and experimental S11 findings are in good agreement.

## 1. Introduction

Researchers have progressively tried to utilize the millimeter-wave (mm-wave) frequency spectrum in several wireless applications in the last few years, including communication systems such as, for instance, 5th generation (5G) communication systems, utilizing mm-wave. Furthermore, the IEEE 802.11ad WiGig and vehicular radar systems incorporate the mm-wave spectrum [1]. The availability of the mm-wave frequency spectrum provides numerous advantages such as wider bandwidth, multi-gigabit data transfer, and a decrease in wavelength offers miniaturization of radiofrequency devices together with the antenna [2,3]. Modern electronic systems such as smartphones and smart devices demand compactness, and this requirement, by default, is fulfilled by the devices operating at the mm-wave spectrum. In the upcoming 5G (mm-wave) era, ultra-low latency and huge data rates entail a reduction in the cost and complication of the transceiver device [4].

The International Telecommunication Union (ITU) has designated a mm-wave frequency range for 5G, extending from 24 GHz to 80 GHz. The operational bands under consideration for 5G mobile networks include 28 GHz, 38 GHz, 60 GHz, and 70 GHz. On the other hand, the 28 GHz and 38 GHz bands are useful for cellular networks, but operating on higher frequency bands would pose challenges to the antenna-configuration mobile communication systems [5,6]. The antenna is one of the crucial components in the transmission link of the future 5G network. Implementing the technology in a mm-wave is vital as the atmosphere absorbs the waves traveling [7]. This scenario demands high gain antennas. The previous research work in 5G applications reveals that the different antenna types can be used to build the wireless communication system, such as monopole [8], dipole [9], magneto-electric dipole [10], loop [11], antipodal vivaldi [12], fractal [13], planar inverted F [14] and so on. Microstrip patch antennas with essential features such as reduced cost, low profile lightweight, and compatibility with monolithic microwave integrated circuit (MMIC) are often used in mobile communication. Prevalent features and easy fabrication of these antennas are found in several fields including satellite, radar technology, mobile communication, etc. The antenna used in mobile phones must be put inside the devices; therefore, design characteristics must be carefully chosen. The literature in the field of mm-wave frequency discloses that the antenna’s physical size should be essentially small, and the associated wavelength becomes a crucial parameter in a fading environment. In Ref. [15], a compact modified ground plane antenna presents a 26.5–32.9 GHz operating frequency. The ground plane modification helps shift the resonant frequency to the lower side and wide bandwidth. The parasitic patch-based microstrip-fed antenna operating in 34.1–38.9 GHz is reported in Ref. [16], with a directivity variation of 6–8 dBi. In Ref. [17], the author shows a dual-band mm-wave antenna with impedance bandwidths of 26.65–29.2 GHz and 36.95–39.05 GHz. The proposed antenna has a peak gain of 1.27 dBi and 1.83 dBi. The antenna presented in Ref. [18] demonstrated a dual-band patch antenna equipped with a Rogers-5880 substrate for 5G networks for 10.15 GHz and 28 GHz operating frequencies and a bandwidth of approximately 0.278 GHz and 1 GHz, respectively. The authors in [19] describe a dual-band patch antenna comprised of a circular structure and an elliptical slot. This configuration has a resonant frequency of 28 GHz and 45 GHz, demonstrating a bandwidth of 1 GHz and 1.3 GHz. In Ref. [20], the Franklin array element is proposed to operate at two frequency bands, one at 21.5–24.3 GHz and the second at 33.9–36 GHz. To accomplish this, the proposed antenna utilized a 3 × 3 array radiating patch and slotted ground plane, respectively. A triangular shape patch antenna operating in both micro and mm-wave antennas is described in Ref. [21]. The defected circular shape radiator with the modified ground plane is exhibited in Ref. [22]. The suggested antenna structure has an operating frequency of 23–28 GHz and a maximum gain of 5.85 dB. The literature in the field of mm-wave frequency discloses that the antenna’s physical size should be essentially small, and the associated wavelength becomes a crucial parameter in a fading environment. As a result, antenna researchers have difficulty designing a small, low-profile antenna operating at millimeter frequency with high gain and wider impedance bandwidth.

This research offers an extremely small antenna with 5 × 3 mm^2^ dimensions for 5G applications that resonate at 28 GHz and has a larger impedance bandwidth of 15.73% (25.8–30.2 GHz). The arc shape antenna proposed in this paper consists of a circular patch put on an FR4 substrate and a full ground plane at the bottom. The antenna structure is implemented by engraving an elliptical slot onto the radiating patch, and this modification results in a resonance frequency of 28 GHz. The following is the organization of the paper. Section 2 discusses the design methodology of the antenna. Section 3 shows the effect of the antenna’s physical characteristics on its performance. Section 5 presents the results of the experiments. In the final part, concluding remarks are presented.

## 2. Antenna Theory and Design

**Figure 1:**
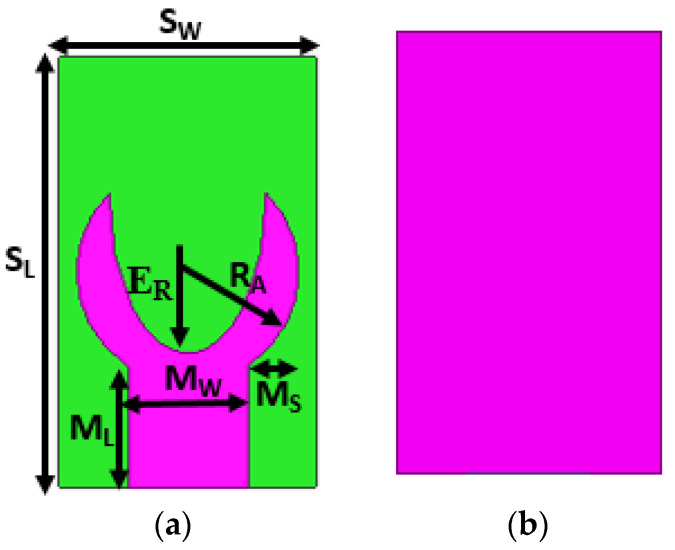
The projected antenna design (**a**) radiating plane (**b**) ground plane.

**Figure 2:**
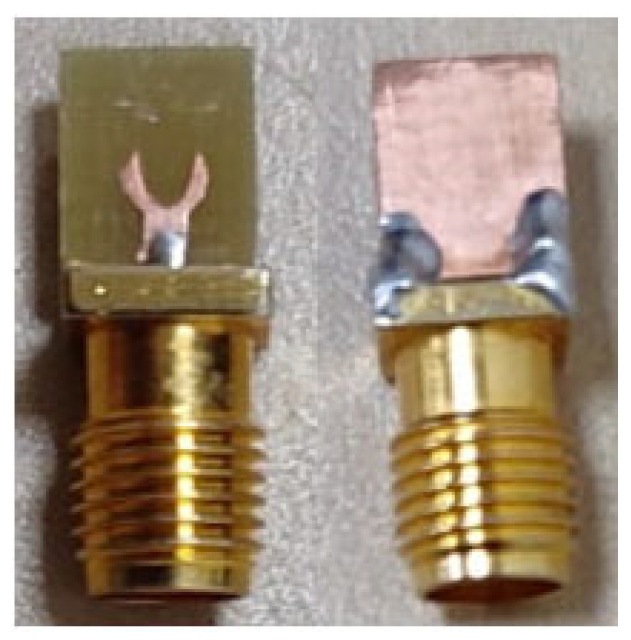
The prototype of the projected antenna design depicts the front and ground plane.

The proposed antenna is designed initially using a circular patch and a complete ground plane with the help of HFSS v13.0. The overall dimensions of the projected antenna are 5 × 3 × 1.6 mm^3^. The circular microstrip patch with a radius R_A_ = 1.3 mm or diameter D_A_ = 2.5 mm, mounted on dielectric substrate material having $\varepsilon_{r}=4.4$, the height of 1.6 mm, and tanδ=0.002. A microstrip feed with dimensions M_L_ = 1.9 mm, and M_W_ = 1.4 mm is used, and a power port is incorporated to 50 Ω. The radius of the proposed arch-shaped patch is calculated using Equation (1). The projected antenna’s design is illustrated in Figure 1. Various antenna parameters were tuned in every design phase to provide the best radiation characteristics. The optimized geometrical parameters of the projected antenna are depicted in Table 1, and a prototype of the same is depicted in Figure 2.

(1) $$R_{A}=\frac{F_{O}}{{\left\{1+\left(\frac{2H}{\pi KF_{O}}\right)\left[ln\left(\frac{\pi F_{O}}{2H}\right)+1.7726\right]\right\}}^{\frac{1}{2}}}$$

The $F_{O}$ is given by Equation (2)

(2) $$F_{O}=\frac{8.791\times 10^{9}}{f\surd K}$$

where K is the dielectric constant of the substrate, *f* is the resonant frequency, and *H* is the thickness of the substrate. For f=28 GHz and K = 4.4, the value of $F_{O}$ is 0.1496. Putting this value of $F_{O}$ in Equation (1), we obtain,

$$R_{A}=\frac{0.1496}{{\left\{1+\left(\frac{2\times 0.16}{\pi \times 4.4\times 0.1496}\right)\left[ln\left(\frac{\pi \times 0.1496}{2\times 0.16}\right)+1.7726\right]\right\}}^{\frac{1}{2}}}=1.2959\;\mathrm{mm}\approx 1.3\;\mathrm{mm}$$

**Figure 3:**
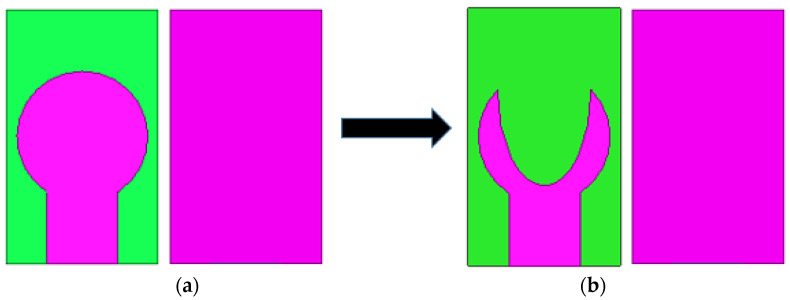
The design development stages of projected antenna (**a**) first stage (**b**) proposed antenna.

**Figure 4:**
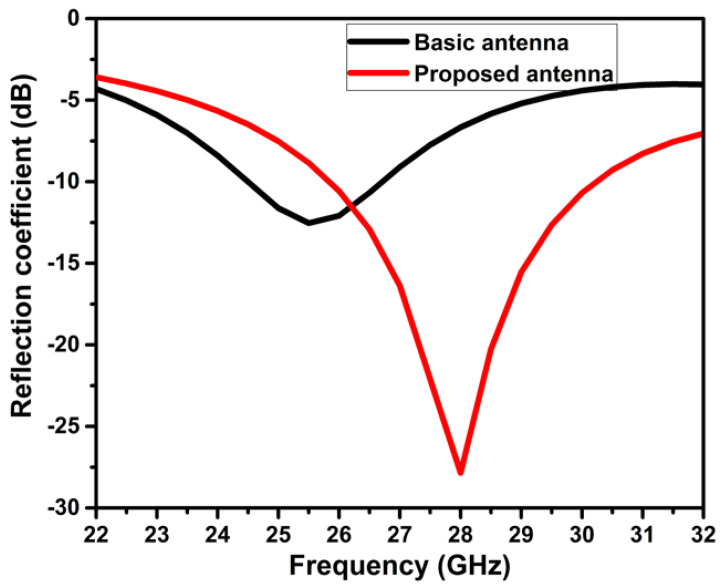
Reflection coefficient of the basic and proposed antenna.

Figure 3 depicts the two primary phases leading to the optimal antenna design. At first, a circular patch is designed using Equation (1) resonating at 25.5 GHz. This antenna has an impedance bandwidth of 24.5 GHz to 26.73 GHz. An elliptical slot is engraved on the circular patch in the subsequent step, as depicted in Figure 3. The incorporation of the slot creates an additional resonating band near the original resonating frequency and increases the bandwidth. The formation of slots significantly impacts the antenna’s far-field radiation and gain. The placement and orientation of the slot on the patch play a vital role in achieving wider bandwidth and increased gain. The position of the elliptical on the radiating plane is tuned, and optimal dimensions are obtained by observing the current circulation on it. The modified antenna exhibits a reflection coefficient well-below −10 dB from 25.8 GHz to 30.2 GHz, as illustrated in Figure 4. The antenna’s gain improved from 3.47 dB to 4.49 dB. The change in antenna parameters in the design evolution process is listed in Table 2.

## 3. Parametric Analysis

A detailed parametric analysis is accomplished to evaluate the effect of the slot on the antenna’s performance. The radius of the circular patch and the elliptical slot determine the design performance. Hence, the parameters *R_A_* and E_R_ are considered.

### 3.1. Effect of Circular Patch R_A_

**Figure 5:**
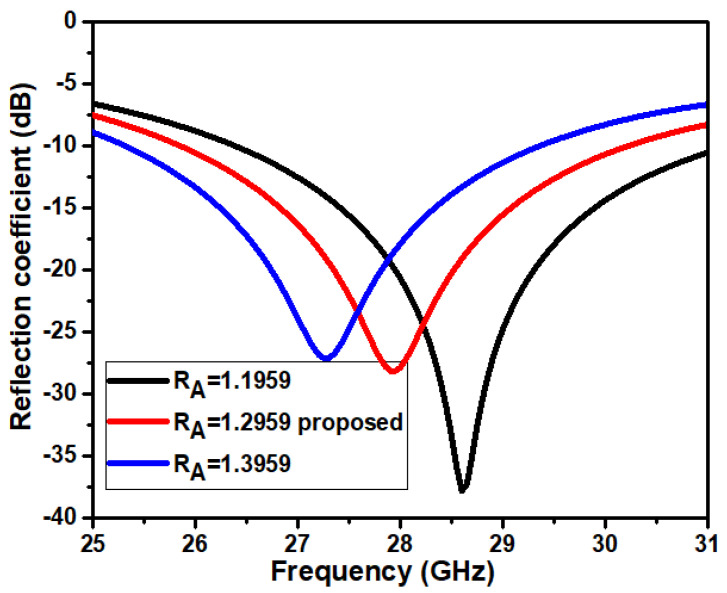
Reflection coefficient concerning *R_A_*.

The impact of circular patch radius *R_A_* is observed on the impedance matching of the antenna. The analysis is carried out by changing the circular patch radius while keeping other dimensions constant. Figure 5 indicates the difference in the reflection coefficient concerning *R_A_*. The simulation results illustrate that the reflection coefficients shift to a different frequency range at every increase in the diameter of the patch. It is witnessed that the variation in the patch radius has a significant effect on reflection coefficients, resonant frequencies, and bandwidth. The values observed for the series of simulations are listed in Table 3. The proposed antenna dimensions (*R_A_* = 1.2959 mm) give the required resonant frequency and broader bandwidth. The proposed antenna has a minimum S11 level of −27.85 dB at 28 GHz and a bandwidth ranging from 25.8 GHz to 30.2 GHz.

### 3.2. Effect of Circular Patch E_R_

**Figure 6:**
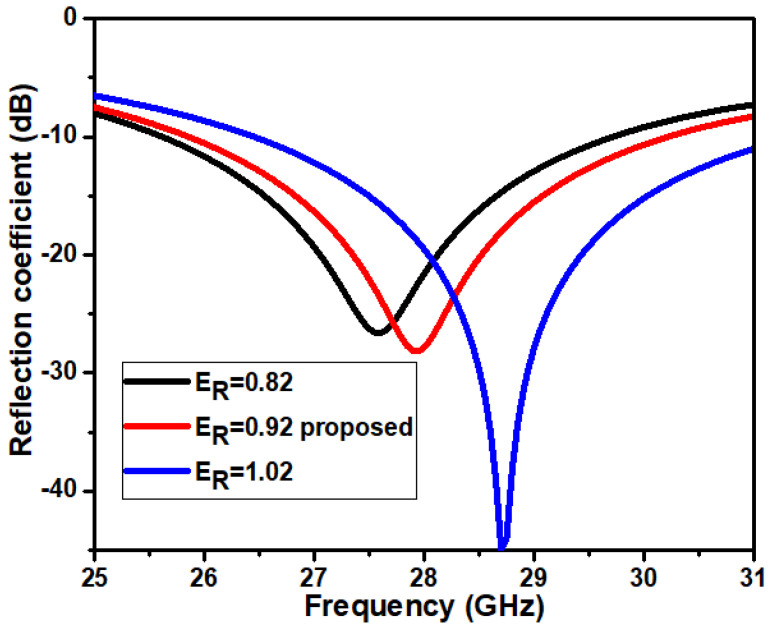
Reflection coefficient concerning E_R_.

The impact of elliptical slot radius E_R_ is observed on the impedance matching of the antenna. The analysis is carried out by changing the elliptical slot radius while keeping other dimensions constant. Figure 6 indicates the difference in the reflection coefficient concerning E_R_. The simulation results illustrate that the reflection coefficient shifts to a different frequency range at every increase in the diameter of the elliptical slot. It is witnessed that the variation in the elliptical slot radius has a significant effect on reflection coefficients, resonant frequencies, and bandwidth. The values observed for the series of simulations are listed in Table 4. The proposed antenna dimensions (E_R_ = 0.92 mm) give the required resonant frequency and broader bandwidth. The proposed antenna has a minimum S11 level of −27.85 dB at 28 GHz and a bandwidth ranging from 25.8 GHz to 30.2 GHz.

## 4. Results and Discussion

This segment depicts an analysis of the proposed antenna performance using several performance measures and a comprehensive comparison of simulated and measured findings.

### 4.1. Return Loss and Current Distribution

**Figure 7:**
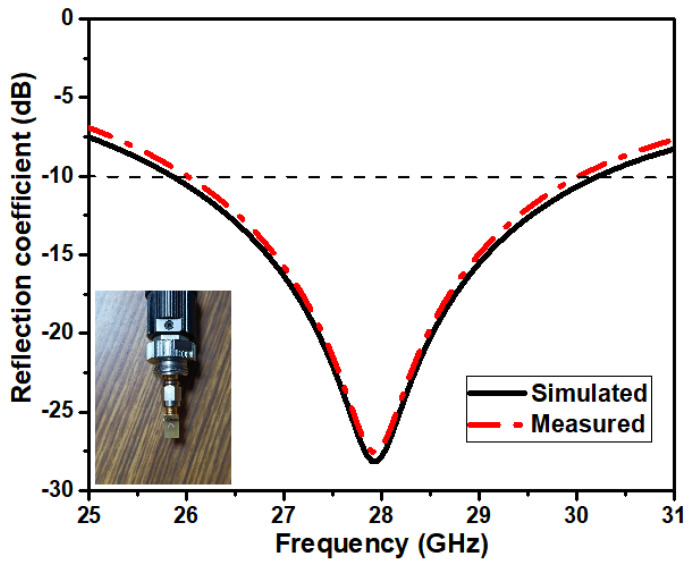
The simulated and measured S11 curve of the designed antenna.

The S11 measures how much power the antenna reflects and is thus known as the reflection (or return loss) coefficient, which is defined in decibels. S11 (dB) must be <−10 dB for the antenna to perform effectively. The suggested antenna has an S11 of −27.85 dB at 28 GHz resonating frequency. The results obtained are optimized by adjusting the diameter values and the elliptical slot locations. Figure 7 illustrates the projected antenna’s simulated and observed reflection coefficient findings. The bandwidth of the proposed antenna structure is 4.4 GHz, ranging from 25.8 GHz to 30.2 GHz. The simulated and experimental findings are consistent and viable for 5G applications.

**Figure 8:**
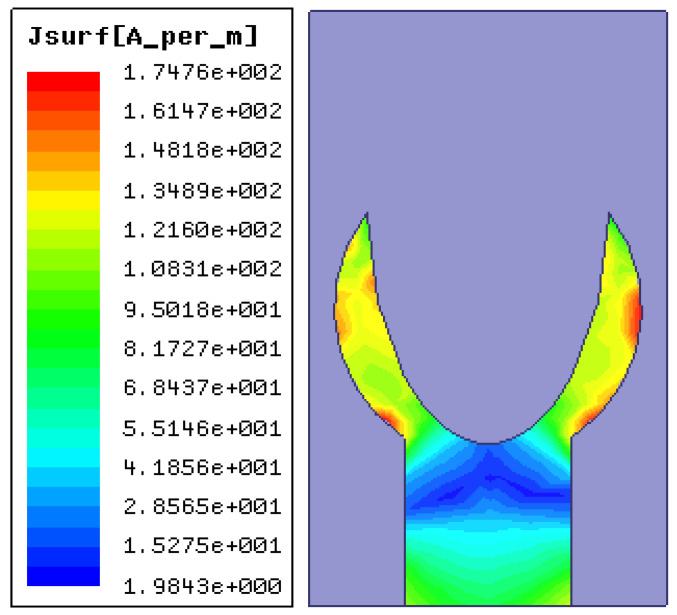
The designed antenna’s current distribution at 28 GHz.

The current distribution of the projected antenna at the resonant frequency is depicted in Figure 8. The maximum current distribution is at the outer edges of the circle. This signifies that the electrically effective length is increased and is evident for the generation of the resonant frequency at 28 GHz.

### 4.2. Radiation Characteristics

**Figure 9:**
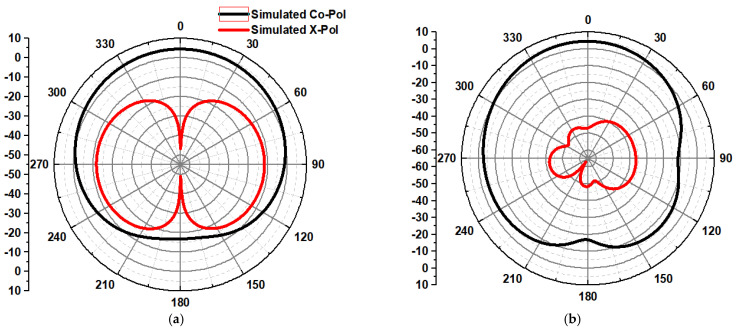
The radiation pattern of the proposed antenna at 28 GHz (**a**) E-plane and (**b**) H-plane.

The radiation pattern of the designed antenna structure at the resonant frequency of 28 GHz is presented in Figure 9. The simulated co- and cross-polarization of two principal planes, E-plane (XZ, ∅ = 0°), exhibit omnidirectional and bidirectional patterns, and H-plane (YZ, ∅ = 90°), exhibit omnidirectional and nearly bidirectional patterns, respectively.

### 4.3. Radiation Characteristics

**Figure 10:**
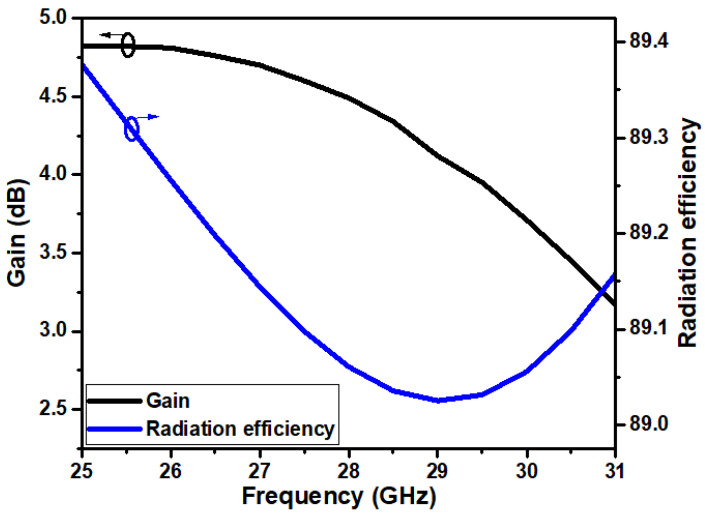
Gian and radiation efficiency of the proposed antenna.

The projected antenna’s simulated gain and radiation efficiency are depicted in Figure 10. The gain of the antenna is almost greater than 4 dB over the impedance bandwidth. At the resonant frequency of 28 GHz, the gain is 4.49 dB. The radiation efficiency of the proposed structure has greater than 89% in the operating frequency.

### 4.4. Comparative Analysis

Table 5 compares the projected antenna to the previously published similar works. The given work offers a miniaturized antenna, compared to the majority of previous works. The proposed antenna provides good radiation characteristics such as larger bandwidth, stable gain, good radiation efficiency, and compacted and straightforward design, which support the design’s applicability for communications systems functioning within the 5G millimeter wave spectrum.

## 5. Conclusions

In this article, a simple and compact microstrip patch antenna with an elliptical slot is designed, developed, and tested at 28 GHz for 5G applications. The structure comprises a circular patch with an elliptical slot to attain the required resonant frequency and a complete ground plane. This simple design is accomplished on an inexpensive FR4 substrate that operates at a frequency of 28 GHz and performs comparatively better than the antennas in the existing literature. According to the measured and simulated findings, the suggested antenna has a broader bandwidth of 4.41 GHz, a high reflection coefficient of −27.85 dB, and a gain greater than 4 dB over the operational band. Furthermore, a comparison of the projected antenna to the previous work demonstrates that the designed antenna is feasible for upcoming wireless communication applications, such as ultra-high definition multimedia, security, and surveillance, that demand a high data rate and more bandwidth.

## Figures and Tables

**Table 1:** Geometrical dimensions of the proposed antenna (mm).

S_L_	S_W_	R_A_	M_L_	M_W_	M_S_	E_R_
5	3	1.3	1.9	1.4	0.7	0.9

**Table 2:** Antenna parameters in the design evolution process.

Evolution Stages	Frequency (GHz)	Bandwidth (GHz)	S11 (dB)	Gain (dB)
**First**	25.5	2.23	−12.54	3.47
**Proposed**	28	4.41	−27.85	4.49

**Table 3:** Antenna simulation results of patch parameter (*R_A_*).

Parameters	Resonant Frequency (GHz)	Bandwidth (GHz)	S11 (dB)
*R_A_* = 1.1959	28.5	4.5	−33.46
*R_A_* = 1.2959 Prop	28	4.41	−27.85
*R_A_* = 1.3959	27.5	4.1	−24.73

**Table 4:** Antenna simulation results of patch parameter (E_R_).

Parameters	Resonant Frequency (GHz)	Bandwidth (GHz)	S11 (dB)
E_R_ = 0.82	27.5	4	−24.46
E_R_ = 0.92 Prop	28	4.41	−27.85
E_R_ = 1.02	28.7	4.8	−45

**Table 5:** Comparative analysis of the proposed antenna and antennas in the existing literature.

References	Resonating Frequency (GHz)	Bandwidth (GHz)	Antenna Dimensions (mm^2^)	Peak Gain (dB)
[6]	28	3.89	15 × 10	5.9
[13]	28	3.76	8 × 5	3.12
[15]	28	6.4	5 × 5	5.6
[17]	28	2.6	14 × 12	1.27
[19]	28	1.3	6 × 6	-
[21]	28	5	30 × 30	5.8
[23]	28	2.3	8 × 8	6.6
[24]	28	4.5	8 × 10	4.25
[25]	28	4.10	5.5 × 4.35	5.3
Proposed Antenna	28	4.41	5 × 3	4.49

## Data Availability

Not applicable.
